# Analysis of Tumor Angiogenesis and Immune Microenvironment in Non-Functional Pituitary Endocrine Tumors

**DOI:** 10.3390/jcm8050695

**Published:** 2019-05-16

**Authors:** Mizuto Sato, Ryota Tamura, Haruka Tamura, Taro Mase, Kenzo Kosugi, Yukina Morimoto, Kazunari Yoshida, Masahiro Toda

**Affiliations:** Department of Neurosurgery, Keio University School of Medicine, 35 Shinanomachi, Shinjuku-ku, Tokyo 160-8582, Japan; mizuto.sato@gmail.com (M.S.); moltobello-r-610@hotmail.co.jp (R.T.); rovin124th@gmail.com (H.T.); mstr.komed05241996@gmail.com (T.M.); kensan03977@yahoo.co.jp (K.K.); yukinaxnashiko@yahoo.co.jp (Y.M.); kazrmky@keio.jp (K.Y.)

**Keywords:** pituitary neuroendocrine tumors, VEGF, Treg, TAM, PD-1, PD-L1

## Abstract

Cavernous sinus (CS) invasion is an aggressive behavior exhibited by pituitary neuroendocrine tumors (PitNETs). The cause of CS invasion in PitNETs has not been fully elucidated. The tumor immune microenvironment, known to promote aggressive behavior in various types of tumors, has not been examined for PitNETs. Vascular endothelial growth factor (VEGF)/VEGF receptor (VEGFR) signaling is strongly associated with the tumor immune microenvironment. In the present study, these molecular and histopathological characteristics were examined in invasive non-functional PitNETs (NF-PitNETs). Twenty-seven patients with newly diagnosed NF-PitNETs (with CS invasion: 17, without CS invasion: 10) were analyzed by immunohistochemistry for VEGF-A/VEGFR1 and 2, hypoxia-inducible Factor (HIF), tumor-infiltrating lymphocytes, immunosuppressive cells including regulatory T cells (Tregs) and tumor-associated macrophages (TAMs), and immune checkpoint molecules. Previously validated tumor proliferation markers including mitotic count, Ki-67 index, and p53 were also analyzed for their expressions in NF-PitNETs. VEGF-A and VEGFR1 were expressed on not only vascular endothelial cells, but also on tumor cells. The expressions of VEGF-A and VEGFR1 were significantly higher in NF-PitNETs with CS invasion. The number of TAMs and the expression of PD-L1 were also significantly higher in NF-PitNETs with CS invasion than in NF-PitNETs without CS invasion. The high expression of VEGF-A and VEGFR1 and associated immunosuppressive microenvironment were observed in NF-PitNETs with CS invasion, suggesting that a novel targeted therapy can be applied.

## 1. Introduction

Pituitary neuroendocrine tumors (PitNETs) are common intracranial tumors that arise from the pituitary gland [1]. In recent years, the development of transnasal endoscopic surgery has improved the surgical outcomes in patients with PitNETs. However, PitNETs often invade into the surrounding cavernous sinus (CS), making them difficult to remove entirely. Although radiation therapy including gamma knife is performed for residual tumors [2], it is onerous to protect essential structures including the optic nerve and internal carotid artery around the sella turcica.

The vascular endothelial growth factor (VEGF)/VEGF receptor (VEGFR) signaling is a potent activator of angiogenesis that is known to correlate with disease progression and hemorrhage in PitNETs [3,4]. The difference in the status of VEGF/VEGFR signaling remains controversial. Niveiro et al. [3] demonstrated that the lowest protein level of VEGF-A was detected in prolactin-secreting PitNETs and the highest levels were detected in non-functional PitNETs (NF-PitNETs). In contrast, Cristina et al. [4] demonstrated that higher expressions of VEGF-A and VEGFR1 were observed in prolactin-secreting PitNETs than in NF-PitNETs.

Recently, the significance of the programmed cell death-1 (PD-1)/programmed cell death ligand-1 (PD-L1) immune checkpoint system in various types of tumors has received attention [5,6]. Anti-PD-1 and PD-L1 antibodies exerted a highly potent effect in the inhibition of tumor growth in melanoma, non-small lung cancer, and kidney cancer [7,8]. Among immune cell types of note, M2 macrophages produce growth factors and anti-inflammatory cytokines to suppress the host immune response [9,10,11]. Tumor-associated macrophages (TAMs) typically behave as M2 macrophages in the tumor immune microenvironment to induce immunosuppression [12,13,14]. Regulatory T cells (Tregs) also exert immunosuppression, resulting in the failure of cancer immunotherapy [15,16]. High Foxp3(+) Tregs infiltration was significantly associated with shorter overall survival in most patients with solid tumors including melanomas and cervical, renal, and breast cancers [17]. VEGF-A plays a pivotal role in the development of these immunosuppressive microenvironments by inhibiting the maturation of dendritic cells and stimulating the proliferation of Tregs [18,19]. However, these immunosuppressive microenvironments have not been fully elucidated in PitNETs.

In the present study, VEGF-A/VEGFRs expressions, the tumor immune microenvironment, and their cross interaction were evaluated, leading to the development of novel treatment strategies for patients with NF-PitNETs.

## 2. Materials and Methods

This research was approved by the Institutional Review Board of our institute (Reference number: 20050002). Written informed consent was obtained from all patients.

### 2.1. Study Population

From April 2011 to October 2017, a total of 27 patients with newly diagnosed NF-PitNETs were analyzed in the present study. All patients received neurosurgical procedures, for mass reduction or diagnostic biopsy, and did not receive radiochemotherapy before the operations.

### 2.2. Immunohistochemical Analysis

Histopathological analyses were performed on 3 μm sections of formalin-fixed paraffin-embedded sections of 27 tumors from 27 patients with newly diagnosed NF-PitNETs that were determined on the basis of the hormonal status in the peripheral blood. NF-PitNETs are usually soft and easy to remove via aspiration. A small amount of tissue was used for pathology assessment. In the present study, a large size of tissue was selected because the multiple, most vascularized regions (hot spots) should be screened for regionally averaged positive cell counts. Mitotic activity was assessed using hematoxylin and eosin (H&E) staining. Immunohistochemistry was performed according to standard procedures [20]. After tissue sections were deparaffinized and rehydrated, antigen retrieval was performed in citrate buffer (Ki-67, p53, VEGFR1, CD34, Foxp3, CD163, CD3, CD4, and PD-1), or in Tris buffer (pH 9 for VEGF-A, VEGFR2, CD8, HIF-1α, and PD-L1) using microwave irradiation or autoclave (HIF-1α and PD-L1). The sections were blocked for 60 min in 2.5% horse serum (ImmPRESSTM Detection Systems, Vectorlabs, CA, USA). The sections were incubated overnight at 4 ℃ with anti-Ki-67 antibody (1:200, M7249, DAKO), anti-p53 monoclonal antibody (1:100, DO-7, DAKO), anti-VEGF-A antibody (1:200, JH121, Merck Millipore), anti-VEGFR1 antibody (1:200, AF321, R&D SYSTEMS), anti-VEGFR2 antibody (1:600, 55B11, Cell Signaling Technology), anti-CD34 antibody (1:100,℃ F1604, Nichirei Biosciences Inc.), anti-Foxp3 antibody (1:100, ab54501, Abcam), anti-CD163 antibody (1:100, ab87099, Abcam), anti-CD3 antibody (1:100, ab5690, Abcam), anti-CD4 antibody (1:200, 1F6, Nichirei Bioscience Inc.), anti-CD8 antibody (1:50, ab17147, Abcam), anti-hypoxia-inducible factor-1α (HIF-1α) antibody (1:100, H-206, Santa Cruz Biotechnology), anti-PD-1 antibody (1:50, NAT105, Abcam), and anti-PD-L1 antibody (1:500, 28-8, Abcam), then incubated with anti-mouse, anti-rabbit, or anti-goat Ig secondary antibody (ImmPRESSTM Detection Systems, Vectorlabs) for 60 min at room temperature. The products were visualized with a peroxidase-diaminobenzidine reaction.

For the assessment of Ki-67 index, manual counting of 1000 tumor cells was routinely done at a high-power field (HPF: ×40) [21]. The positivity of VEGF-A staining in the tumor cytoplasm or stroma was assessed as the following: ++, diffuse intense staining; +, diffuse faint staining; −, negative staining. The staining positivity of VEGFR1 and VEGFR2 on endothelial cells was assessed as the following: +, staining in vascular endothelial cells; −, negative staining. For the assessment of microvessel density (MVD), the tissue sections were screened at low-power fields (×4), and the three most vascularized regions (hot spots) were selected for each region. The counting of microvessels was performed on these regions at HPFs (×20, 0.95 mm^2^). HIF-1α expression was assessed as the following: ++, expression in >10% of tumor cells; +, expression in ≤10% of tumor cells; −, negative staining [22]. For the assessment of density of Foxp3, CD163, CD4, and CD8 (+) cells, the tissue sections were screened using each immunohistochemistry at the low-power fields (×4), and three hot spots were selected. Counting of the positive cells was performed in these areas at the HPFs (×40, 0.47 mm^2^). PD-L1 expression was assessed as the following: 3+, expression in ≥50% of tumor cells; 2+, expression in ≥5% and <50% of tumor cells; 1+, expression in ≥1% and <5% of tumor cells; 0, expression in <1% of tumor cells [23]. Both histopathological reviewing and scoring were independently performed with blinded clinical information by three authors (MS, RT, and YM).

The specificity of immunohistochemistry was checked using negative and positive controls. For negative controls, paraffin sections were incubated with non-immune mouse, rabbit, and goat IgG at the same concentration used for each antibody. Sections from glioblastomas were used as the positive controls for each antibody (Figure S1).

### 2.3. Radiographical Analysis

The existence of CS invasion was evaluated by gadolinium (Gd)-enhanced T1-weighted images. We classified NF-PitNETs into two types: NF-PitNETs with CS invasion and NF-PitNETs without CS invasion. Cystic formation and hemorrhage components were evaluated using T1- or T2-weighted images. Tumor size was volumetrically measured via Gd-enhanced imaging, as previously described [20].

### 2.4. Statistical Analysis

Student’s *t*-test was used for the quantitative analysis of Ki-67, mitotic count, Foxp 3, CD 163, PD-1, CD 4, and CD 8 (+) cells and the ratio of Foxp3 (+) cells to CD8 (+) cells in the CS (+) group and the CS (−) group. For the scores of VEGF-A, VEGFR1, VEGFR2, HIF-1α, and p53 the chi-squared test was used. PD-L1 expression on tumor cells was scored according to the percentage of PD-L1 positive cells (score 0–4). Therefore, nonparametric analysis of Mann-Whitney U-test was used to test the immunostaining raw scores of PD-L1 expression between the two groups, considering that the analytical immunohistochemistry scores were not normally distributed. All statistical analyses were performed using IBM SPSS statistics (IBM Corp., Armonk, NY, USA). A *p*-value of <0.05 was considered statistically significant.

## 3. Results

### 3.1. Patients’ Characteristics

**Figure 1:**
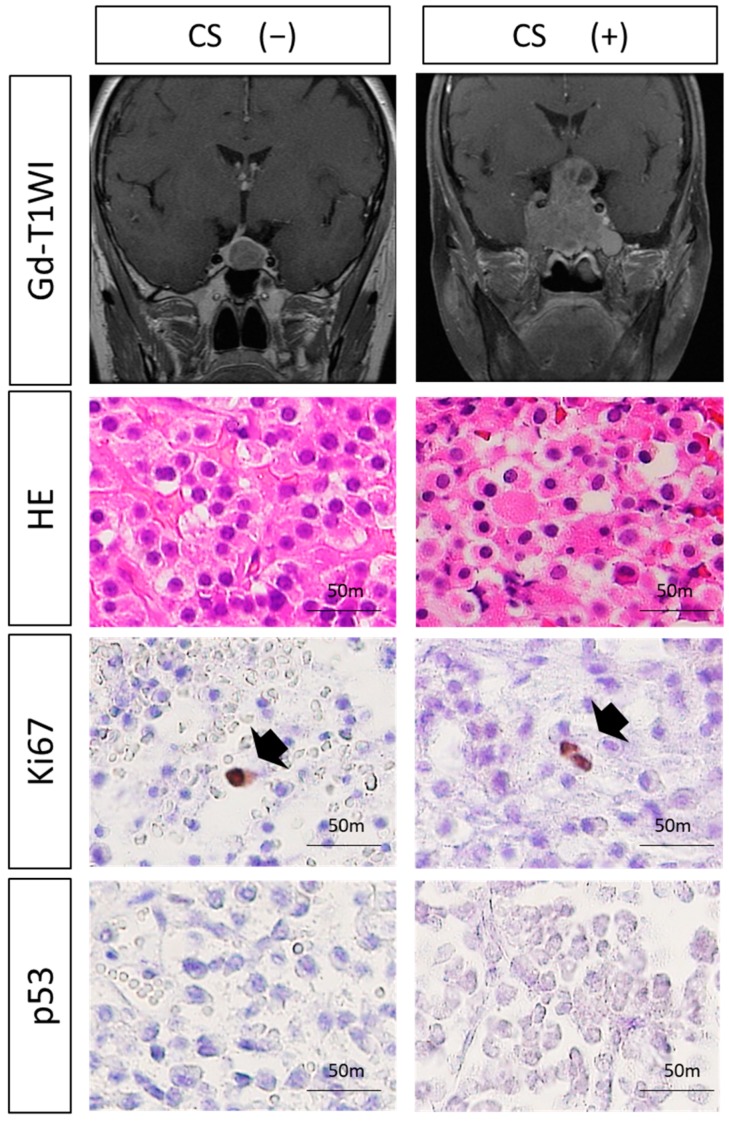
Analysis of classical histological atypical features for invasive non-functional pituitary neuroendocrine tumors (NF-PitNETs). The existence of CS invasion was evaluated by gadolinium (Gd)-enhanced T1-weighted images. There were no significant differences in Ki-67 and p53 expression or mitotic count between NF-PitNETs with CS invasion and NF-PitNETs without CS invasion (Ki-67 and mitotic count, student’s *t*-test; p53, chi-squared test). Black arrow: tumor cell showing positive Ki-67 expression (Original magnification, ×20).

Characteristics of 27 patients with newly diagnosed NF-PitNETs are summarized in Table 1. The patients were categorized into a CS (+) group (*n* = 17) and a CS (−) group (*n* = 10) (Figure 1, Table 1). The average age of patients with NF-PitNETs exhibiting CS invasion was higher than in those without CS invasion (*p* = 0.0030). There was no significant difference in terms of sex in both groups (*p* = 0.45). Tumor volume was significantly higher in the CS (+) group than in the CS (−) group (*p* = 0.0011). However, some NF-PitNETs easily invade into the CS despite their small tumor size. There were no significant differences between the two groups in cystic formation (*p* = 0.78) and hemorrhagic component (*p* = 0.89).

### 3.2. Histological Analysis

No significant differences were observed in mitotic count between the two groups (*p* = 0.38) (Figure 1, Table 1). Ki-67 index was <1%, and p53 was immunonegative in all patients (Figure 1, Table 1).

### 3.3. Expressions of VEGF-Related Molecules and MVD

**Figure 2:**
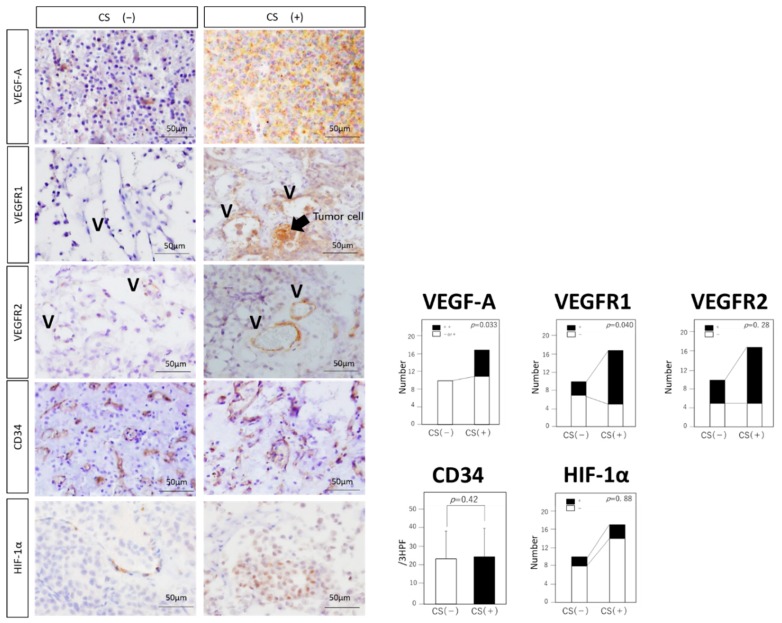
Expressions of VEGF-A related molecules in NF-PitNETs. Immunohistochemical analysis of VEGF-A, VEGFR1, VEGFR2, CD34, and HIF-1. Typical examples of each staining are shown in both groups. Black arrow: tumor cells showing positive VEGFR1 expression. V: vascular structure (original magnification, ×20). Statistical analysis of each staining is shown. Expressions of VEGF-A and VEGFR1 are significantly higher in the CS (+) group than in the CS (−) group (VEGF-A: *p* = 0.033, VEGFR1: *p* = 0.040). Expressions of VEGFR2 and HIF-1α do not reach statistical significance (VEGFR2: *p* = 0.28, HIF1-α: *p* = 0.88). MVD shows no significant difference between the two groups (*p* = 0.42). Data represent the mean ± standard error of mean (VEGF-A, VEGFR1, VEGFR2 and HIF-1α, chi-squared test; MVD, student’s *t*-test).

Expressions of VEGF-A and VEGFR1 were significantly higher in the CS (+) group than in the CS (−) group (VEGF-A: *p* = 0.033, VEGFR1: *p* = 0.04) (Figure 2). VEGFR2 expression showed no significant difference between the two groups (*p* = 0.28). VEGF-A and VEGFR1 were expressed on not only endothelial cells, but also on tumor cells. MVD showed no significant difference between the two groups (*p* = 0.42; Figure 2), and the average of all cases in both groups, 24.9/3HPF, was equivalent to that of other central nervous tumors with high vasculatures, previously described [19]. Expression of HIF-1α showed no significant difference between the two groups (*p* = 0.88; Figure 2).

### 3.4. Tumor-Infiltrating Immune Cells

**Figure 3:**
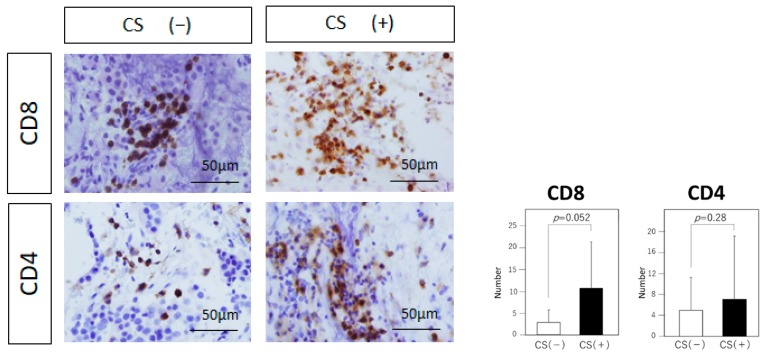
Analysis of tumor-infiltrating lymphocytes. Immunohistochemical analysis of CD8 and CD4 (Original magnification, ×20). Typical examples of each staining are shown in both groups. Statistical analysis of each staining is shown. The number of CD8 (+) lymphocytes tends to be higher in the CS (+) group than in the CS (−) group, but the difference is not statistically significant (*p* = 0.052). The number of CD4 (+) lymphocytes shows no significant difference between the two groups (*p* = 0.28). Data represent the mean ± standard error of mean (CD4 and CD8, student’s *t*-test).

**Figure 4:**
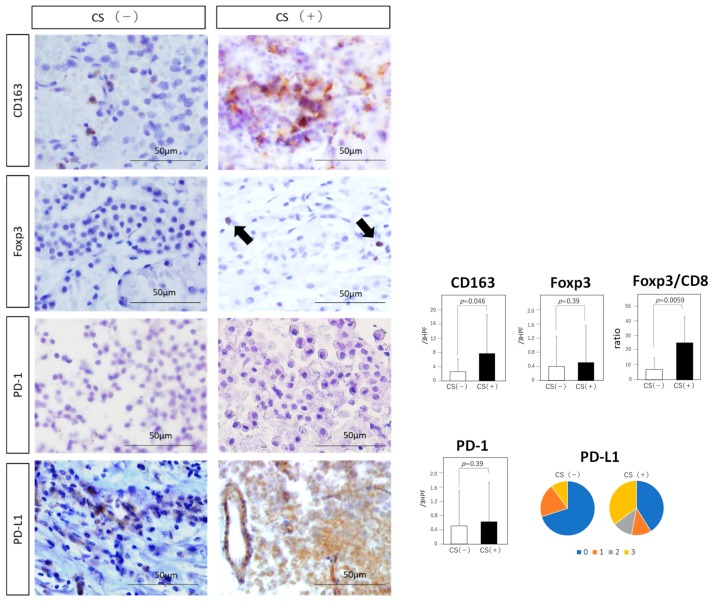
Analysis of immunosuppressive cells and immune checkpoint molecules. Immunohistochemical analysis of CD163, Foxp3, PD-1, and PD-L1 (Original magnification, ×20). Typical examples of each staining are shown in both groups. Black arrow: tumor cell showing positive Foxp3 expression. The number of CD163 (+) tumor-associated macrophages (TAMs) and Foxp3/CD8 ratio are significantly higher in the CS (+) group than in the CS (−) group (CD163: *p* = 0.046, Foxp3/CD8: *p* = 0.0059). The score of PD-L1 tends to be higher in the CS (+) group than in the CS (−) group (*p* = 0.050). Expressions of Foxp3 and PD-1 do not reach statistical significance (Foxp3: *p* = 0.39, PD-1: *p* = 0.39). Data represent the mean ± standard error of mean (CD163, Foxp3, PD-1 and Foxp3/CD8 ratio, student’s *t*-test; PD-L1, Mann-Whitney *U* test).

The number of CD8 (+) lymphocytes tended to be higher in the CS (+) group than in the CS (−) group, but the difference is not statistically significant (10.81 vs. 2.9, *p* = 0.052; Figure 3). The number of CD4 (+) lymphocytes showed no significant difference between the two groups (6.94 vs. 4.89, *p* = 0.28; Figure 3). The number of immunosuppressive CD163 (+) cells was significantly higher in the CS (+) group than in the CS (−) group (7.7 vs. 2.6, *p* = 0.046; Figure 4). Although the number of immunosuppressive Foxp3 (+) cells showed no significant difference between the two groups (0.5 vs. 0.4, *p* = 0.39; Figure 4), Foxp3/CD8 ratio was significantly higher in the CS (+) group than in the CS (−) group (25.87 vs. 7.25, *p* = 0.0059; Figure 4).

### 3.5. Immune Checkpoint Molecules

The expression of PD-L1 was observed on cell membrane and in the cytoplasm of tumor cells (Figure 4). The endothelial cells were also occasionally immunopositive for PD-L1. In the CS (+) group, the PD-L1 score was 2 or 3 in eight patients, and 0 or 1 in nine of the 17 patients. In contrast, in the CS (−) group, the PD-L1 score was 2 or 3 in one patient, and 0 or 2 in nine of the 10 patients. The score tended to be higher in the CS (+) group than in the CS (−) group, but the difference is not statistically significant (*p* = 0.050; Figure 4). There were no significant differences in PD-1 (+) cells between the two groups (0.61 vs. 0.50, *p* = 0.39).

## 4. Discussion

CS invasion is a commonly demonstrated aggressive behavior exhibited by PitNETs [24,25,26], and this property has been recommended to describe aggressive PitNETs in the revised 2017 World Health Organization (WHO) classification [1]. Recently, Rutkowski et al. [27] re-emphasized the importance of classical histological characteristics. They demonstrated that mitotic activity, extensive p53 staining, and Ki-67 index were associated with poor prognosis [27]. However, in the present study, these classical histological characteristics did not show a correlation with CS invasion.

In contrast, our data suggested that VEGF-A/VEGFR1 expressions could be associated with CS invasion. The relationship between the expressions of VEGF-A/VEGFR1 and the prognosis of PitNETs has been previously discussed [28,29]. VEGF-A and VEGFR1 are known to contribute to the tumor cell growth of PitNETs [28,30,31]. Some studies have demonstrated that VEGFR2 is widely expressed in NF-PitNETs, with aggressive behavior such as suprasellar extension in NF-PitNETs [3,32]. MVD, characterized by CD31 immunopositivity and VEGF-A expression, reflected poor prognosis of NF-PitNETs [4]. Our findings corroborate with the findings of these studies. Importantly, VEGF-A and VEGFR1 were expressed on not only endothelial cells, but also on tumor cells, which have been previously confirmed using PitNETs cell line HP75 [33,34]. Tumor cells expressing VEGFR1 themselves release VEGF-A, and an autocrine regulatory function for VEGF in tumor growth in PitNETs is plausible.

Xiao et al. demonstrated rapid and hemorrhagic transformation in PitNETs via the HIF-1α hypoxic signaling pathway [35]. Interestingly, there was no significant correlation in the expression levels of HIF-1α and VEGF mRNA in PitNETs, although VEGF-A is mainly induced by HIF-1α [35]. RSUME, a small RWD-domain containing protein, was reported to play an important role in tumor neovascularization by regulating VEGF-A production in PitNETs [36,37,38,39]. The lack of correlation between VEGF-A and HIF-1α observed in the present study was in accordance with previous observations [35]. It is noteworthy that Barbagallo et al. [40] demonstrated that circSMARCA5, which acts as circular RNA for the splicing factor Serine and Arginine Rich Splicing Factor 1 SRSF1 in glioblastomas, is an upstream regulator of VEGF-A. Other regulators, such as circSMARCA5, might be involved in the VEGF-A expression of PitNETs.

Other aggressive characteristics, such as cystic change, were previously correlated with upregulated VEGF-A [29]. However, controversy exists over the relationship between hemorrhagic change and VEGF-A expression [29,41]. VEGF-A was not associated with cystic or hemorrhagic change in the present study. The cause for the discrepancy in the status of cystic and hemorrhage change between previous relevant studies and this study remains unclear. It could be attributed to the small sample size, highly heterogeneous PitNETs, and the difference between the analytical methods of immunohistochemistry and quantitative analysis (RT-PCR and western blot). Although VEGF-A is widely considered as a marker of poor prognosis in PitNETs, Takada et al. could not find significant correlations between vascularity and other clinical and endocrinological parameters, suggesting that angiogenesis is not essential for growth or invasiveness of PitNETs [42]. Further analysis using a large number of patients might elucidate the role of VEGF-A in PitNETs.

There is a lack of studies related to the tumor microenvironment of PitNETs. PD-L1 RNA and protein expression were significantly increased in recurrent functioning (growth hormone and prolactin-expressing) PitNETs compared with in NF-PitNETs (null cell and silent gonadotroph). Tumor infiltrating CD8 (+) lymphocytes were positively correlated with increased PD-L1 expression [43,44]. In the present study, most NF-PitNETs without CS invasion showed low PD-L1 expression score and low CD8 (+) lymphocyte count, which was compatible with previous studies [43,44]. However, some NF-PitNETs with CS invasion demonstrated a high PD-L1 expression score and a high number of CD8 (+) lymphocyte counts. Interestingly, PD-1/PD-L1 expressions are known to be associated with VEGF-A exposure [45,46].

Tumor size in NF-PitNETs is positively correlated with the number of CD68+ macrophages [47]. Macrophages express different functional programs in response to microenvironmental signals, which is defined as M1/M2 polarization [48]. CD68 antigen is expressed on both M1 and M2 macrophages, and CD163 is a specific marker for M2 macrophages [48]. Although the number of CD163 + M2 macrophages (TAMs) was not associated with the tumor volume, TAMs were associated with CS invasion in the present study. TAMs produce matrix metalloproteinase (MMP)-9 [48] that might promote the invasive behavior of PitNETs. Furthermore, VEGF-A is known to promote the immunosuppressive microenvironment [49], as well as the migration and differentiation of TAMs from immature myeloid cells [50,51].

Upregulation of VEGF-A induces VEGFR-2-expressing Tregs and also promotes their recruitment to the tumor microenvironment via over-expression of chemokine—chemokine ligand 28 by tumor cells [52]. Foxp3/CD8 ratio are known to correlate with the immunosuppressive microenvironment [46,53]. In the present study, the Foxp3/CD8 ratio was strongly associated with CS invasion, which might serve as a new biomarker of invasive NF-PitNETs.

The results obtained in the present study suggest that VEGF-A/VEGFR1 expression can be a treatment target. Blocking VEGF-A can regulate immunosuppressive cells such as TAMs. However, PitNETS with high PD-L1 expression deserve special attention as they correlate to poor outcomes of certain chemo- and immunotherapies [45,54,55,56,57].

A limitation of this study was the paucity of the number of patients. Other invasive markers such as MMP-9 and -14 were previously correlated with the hemorrhage and invasive behavior of PitNETs [41,58]. Future studies should analyze the role of these MMPs in a large number of patients to confirm the findings of this study. In addition, NF-PitNETs are morphologically heterogeneous. The new classification by WHO in 2017 was based on hormone immunohistochemistry and pituitary transcription factors. Although gonadotroph adenoma is the most common subtype among non-functional adenomas [1,59], some cases with thyroid stimulating hormone (TSH), growth hormone (GH), adrenocorticotropic hormone (ACTH), or prolactin (PRL) stainings behave as silent adenomas with no secretion [1]. The relationship between VEGF/VEGFR signaling, tumor microenvironment, and the above-mentioned hormonal and transcriptional characteristics should be investigated in future studies.

## 5. Conclusions

The high expressions of VEGF-A and VEGFR1 were observed in NF-PitNETs with CS invasion. Immunosuppressive microenvironments including TAMs and immune checkpoint molecules, which are induced by VEGF-A, were also associated with NF-PitNETs with CS invasion.

## Figures and Tables

**Table 1:** Patient characteristics and results.

	CS Invasion (+)	CS Invasion (−)	*p* Value
Number	17	10	-
Age (years old)	66.06 (37–85)	49.45 (32–76)	0.0030
Sex	Male: 6, Female: 11	Male: 5, Female: 5	0.45
Cystic formation	6	3	0.78
Hemorrhagic component	2	1	0.89
Tumor volume (cm^3^)	27.75 ± 22.33	7.16 ± 7.23	0.0011
Ki-67 index	<1%: 17	<1%: 10	-
Mitotic count	0/10HPF: 13 1/10HPF: 3 2/10HPF: 1	0/10HPF: 9 1/10HPF: 1	0.38
p53 IHC positive	0	0	-
VEGF-A expression	++: 6 + or −: 11	++: 0 + or −: 10	0.033
VEGFR1 expression	+: 12 −: 5	+: 3 −: 7	0.040
CD163 expression	7.70 ± 10.9	2.60 ± 3.53	0.046

CS: cavernous sinus, IHC: immunohistochemistry, VEGF: vascular endothelial growth factor, VEGFR: vascular endothelial growth factor receptor.

## Supplementary Materials

The following are available online at https://www.mdpi.com/2077-0383/8/5/695/s1, Figure S1: Positive controls for each antibody.
