# Supplementary material for: Analysis of Tumor Angiogenesis and Immune Microenvironment in Non-Functional Pituitary Endocrine Tumors
Source: J Clin Med. 2019 May 16;8(5):695. doi: 10.3390/jcm8050695 (PMC6572068; doi:10.3390/jcm8050695)
Supplement: Supplementary file 1 [file jcm-08-00695-s001.pdf]

Analysis of Tumor Angiogenesis and Immune Microenvironment in Non-Functional Pituitary Endocrine Tumors

Mizuto Sato, Ryota Tamura, Haruka Tamura, Taro Mase, Kenzo Kosugi, Yukina Morimoto, Kazunari Yoshida and Masahiro Toda *

Supplementary Figure 1

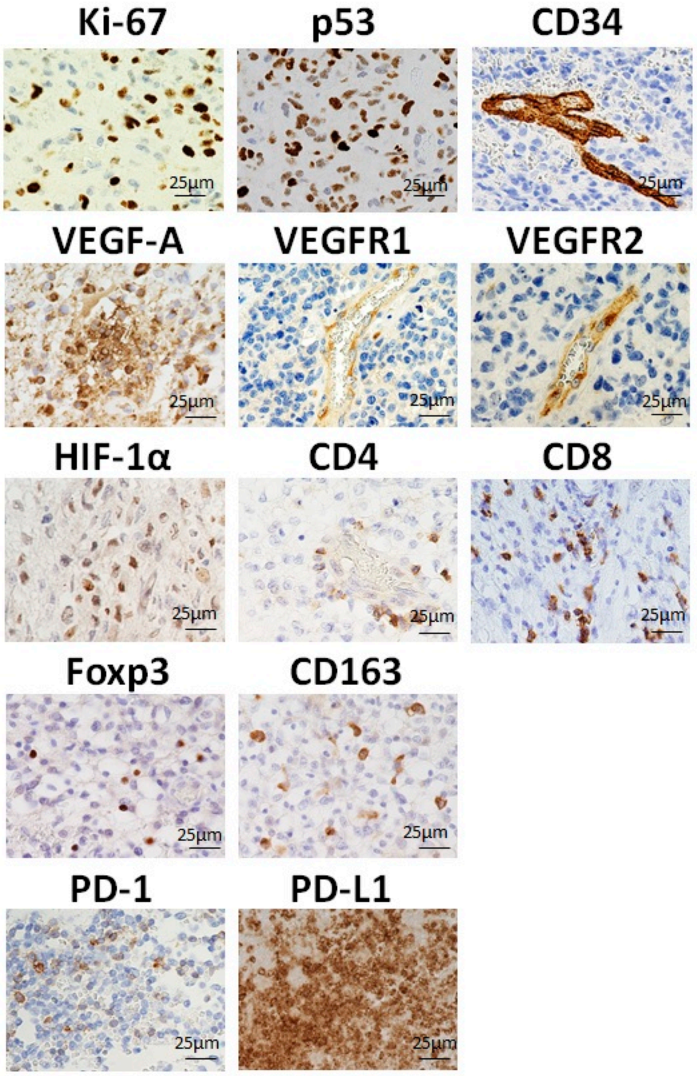

Figure S1. Positive controls for each antibody.
